# Incidence and risk factors of micronutrient deficiency in patients with IBD and intestinal Behçet’s disease: folate, vitamin B12, 25-OH-vitamin D, and ferritin

**DOI:** 10.1186/s12876-021-01609-8

**Published:** 2021-01-21

**Authors:** Yong Eun Park, Soo Jung Park, Jae Jun Park, Jae Hee Cheon, TaeIl Kim, Won Ho Kim

**Affiliations:** 1 Department of Internal Medicine, Yonsei University College of Medicine, 50-1 Yonsei-ro, Seodaemun-gu, Seoul, 03722 Republic of Korea; 2 Institute of Gastroenterology, Yonsei University College of Medicine, Seoul, Republic of Korea; 3 Division of Gastroenterology, Department of Internal Medicine, Inje University College of Medicine, Haeundae Paik Hospital, Busan, Republic of Korea

**Keywords:** Inflammatory bowel disease, Intestinal behçet’s disease, Micronutrients, Risk factors

## Abstract

**Background:**

Patients with inflammatory bowel disease (IBD) and intestinal Behçet’s disease (BD) are vulnerable to micronutrient deficiencies due to diarrhea-related gastrointestinal loss and poor dietary intake caused by disease-related anorexia. However, few studies have investigated the incidence and risk factors for micronutrient deficiency.

**Methods:**

We retrospectively analyzed 205 patients with IBD who underwent micronutrient examination, including folate, vitamin B12, 25-OH-vitamin D, and/or ferritin level quantification, with follow-up blood tests conducted 6 months later.

**Results:**

Eighty patients (39.0%), who were deficient in any of the four micronutrients, were classified as the deficiency group, and the remaining 125 (61.0%) were classified as the non-deficient group. Compared to those in the non-deficiency group, patients in the deficiency group were much younger, had more Crohn's disease (CD) patients, more patients with a history of bowel operation, and significantly less 5-amino salicylic acid usage. Multivariate analysis revealed that CD and bowel operation were significant independent factors associated with micronutrient deficiency.

**Conclusions:**

The incidence of micronutrient deficiency was high (39.0%). Factors including CD, bowel operation, and younger ages were found to be associated with higher risks of deficiency. Therefore, patients with IBD, especially young patients with CD who have undergone bowel resection surgery, need more attention paid to micronutrition.

## Background

Inflammatory bowel disease (IBD), including Crohn's disease (CD) and ulcerative colitis (UC), is a chronic disease of the gastrointestinal (GI) tract associated with unclear etiology, leading to rectal bleeding, abdominal pain, and weight loss and repeated cycles of relapse and remission [1, 2]. CD is an IBD that can affect entire GI tract from mouth to anus and it affects mainly terminal ileum and colon. It often includes both intestinal and extra-intestinal symptoms [2, 3]. On the other hand, UC is mostly restricted to the colon and it usually involves continuous lesion in the intestinal mucosa [2]. Most patients with IBD, especially those with CD, suffer from weight loss and malnutrition during the course of the disease [4, 5], which may be related to lack of oral intake, increased nutrient requirements, increased GI loss, and intestinal resection or bypass surgery [4, 6]. In addition, intestinal Behçet’s disease (BD), which is an intestinal invasion of BD with chronic relapsing multisystem vasculitis disorder [7], is similar to CD with respect to clinical courses, symptoms and treatment modalities [8]. Therefore, there is increasing interest in patient management and nutritional status in intestinal BD as well as IBD.

Nutrients can be classified as either macronutrients or micronutrients. Macronutrients are energy-providing nutrients including carbohydrates, lipids, and proteins. Malnutrition can occur in cases of active, severe IBD, when macronutrients are not consumed or absorbed in sufficient quantities. Micronutrients, including minerals, vitamins, and trace elements, are often deficient in patients with mild disease activity or remission status [9, 10].

According to the European Society for Clinical Nutrition and Metabolism guidelines, patients with IBD should be regularly checked for micronutrient deficiencies and certain deficits should be adequately corrected [11]. Several studies have reported vitamin and mineral deficiencies in patients with IBD; these studies assessed their symptoms and effects on the quality of life and observed widely variable clinical significance [12–14]. Vitamins are organic compounds and are classified as either water-soluble, including thiamine (B1), riboflavin (B2), nicotinic acid/niacin (B3), pyridoxine (B6), cobalamin (B12), biotin, pantothenic acid, folic acid, and vitamin C (ascorbic acid), or fat-soluble, including vitamins A, D, E, and K [9]. Dietary minerals are important inorganic components that work as cofactors and catalysts in maintaining cell structure and enzymatic processes, such as calcium, phosphate, potassium, magnesium, and iron. Trace elements are necessary for the function of enzymes in the body, including zinc, copper, and selenium [9, 10].

Clinically relevant micronutrient deficiencies that occur over the course of IBD disease progression include anemia (caused by iron, folate, and vitamin B12 deficiencies), bone mineral density loss (due to insufficient calcium, vitamin D, magnesium, and vitamin K levels), impaired thrombosis (caused by folate, vitamin B6, B12 deficiency) and wound healing deficits (due to deficiencies of vitamin A, C, and zinc), and carcinogenesis (related to folate, vitamin D, and calcium deficiency) [9]. Among them, anemia is the most common complication affecting up to 70% of patients with IBD, including UC and CD, and intestinal BD [15, 16]. Iron deficiency is the most common cause of anemia in 30–90% of patients with IBD, but folate and vitamin B12 deficiencies are also highly prevalent in these patients, especially in those with CD, compared to the general population [17–19]. In addition, bone density is an important factor, which affects not only the quality of life of IBD patients but also the disease course of IBD, as it is highly related to treatment modalities such as corticosteroids as well as micronutrients [20–22]. However, studies assessing micronutrient concentrations in patients with IBD are scarce, and there are currently no studies on micronutrients in patients with intestinal BD, to our knowledge. Therefore, we aimed to investigate the prevalence and risk factors of micronutrient deficiency in patients with IBD and intestinal BD.

## Methods

### Patients

**Fig. 1:**
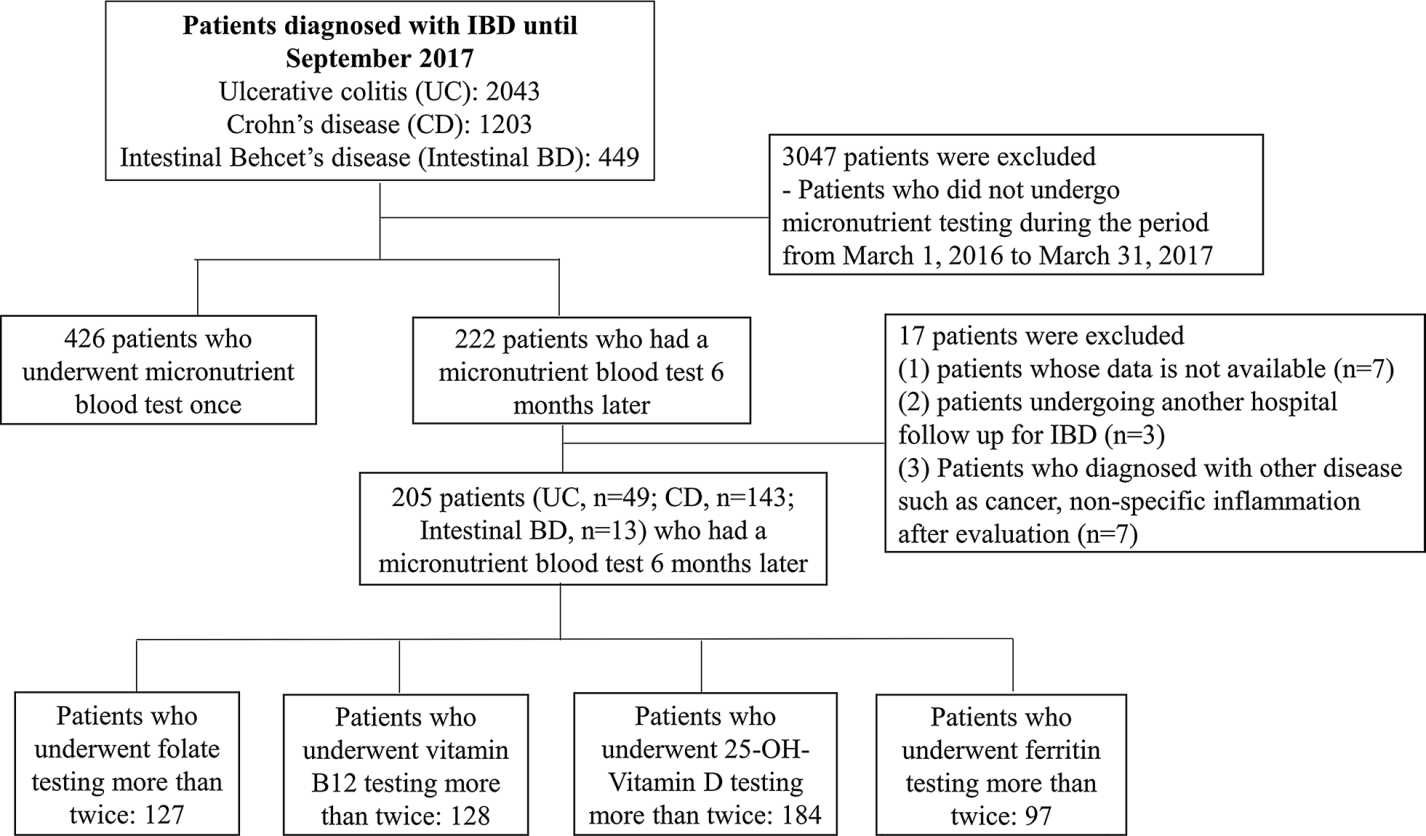
Patients enrollment

We conducted a retrospective study of patients with IBD and intestinal BD who underwent laboratory tests to quantify micronutrients such as iron, folate, vitamin B12, and 25-OH-vitamin D from March 2016 to March 2017 at the Severance Hospital, Yonsei University College of Medicine, Seoul, Korea. Out of 3,695 patients, a total of 205 with IBD and intestinal BD who underwent micronutrient testing twice were enrolled retrospectively. A total of 3,490 patients were excluded from our study for the following reasons: (1) patients did not undergo micronutrient testing during the study period (n = 3,047); (2) patients underwent micronutrient blood testing only once (n = 426); (3) data were not available or were lost to follow-up (n = 10); and (4) patients diagnosed with other diseases such as cancer or non-specific inflammation after evaluation (n = 7) (Fig. 1).

We included patients who underwent micronutrient testing at least twice, at baseline and at follow-up after 6 months for one or more of the following four micronutrients: folate (n = 127), vitamin B12 (n = 128), 25-OH-vitamin D (n = 184), and ferritin (n = 97). In addition, we divided patients into two groups: a group consisting of those with a micronutrient deficiency (n = 80) and another group with those without micronutrient deficiency (n = 125) at baseline. This study was performed in accordance with the ethical guidelines of the 1975 Declaration of Helsinki and was approved by the institutional review board of Severance Hospital.

### Baseline characteristics

Variables of baseline characteristics included demographic information, routes of laboratory tests (outpatients or inpatients), medications, supplements, past bowel surgery, and underlying diseases.

### Definitions

We defined deficient levels of folate and vitamin B12 as $<\hspace{0.17em}$3.38 ng/mL (folate N: 3.38–48.0 ng/mL) and < 211 pg/mL (vitamin B12 N: 211–911 pg/mL), respectively. Iron deficiency was defined as ferritin levels < 10 ng/mL (N: 10–291 ng/mL). Serum 25-OH-vitamin D testing was performed to identify vitamin D deficiency, with vitamin D deficiency defined as < 10 ng/mL (50 nmol/L).

In our study, we defined bowel surgery as cases of surgery due to diseases of IBD and intestinal BD only, and excluded all the other causes including diverticulum and foreign body.

### Statistical analysis

Variables are expressed as either median (interquartile range, IQR) or n (%). Baseline characteristics were compared using independent Student’s t-tests (or Mann–Whitney tests) for continuous variables, and χ^2^ tests (or Fisher’s exact tests) were used for categorical variables, as appropriate. Independent predictors of micronutrient deficiency were analyzed using logistic regression analysis. Odds ratios (ORs) and the corresponding 95% confidence intervals (CIs) were calculated. Data analysis was performed using Statistical Package for the Social Sciences (SPSS) software (version 20.0; SPSS Inc., Armonk, NY, USA). A *P-*value < 0.05 was considered statistically significant.

## Results

### Patient characteristics

Micronutrient deficiency was observed in 80 (39.0%) of 205 patients who were tested over the year beginning March 2016. Baseline characteristics of the micronutrient deficient and non-deficient groups were analyzed and are summarized in Table 1. Among the micronutrients assessed, 25-OH-vitamin D was quantified in 90.2% of patients, and vitamin B12, folate and ferritin testings were performed for 47.3–62.4% of patients. The proportions of folate (80.0% vs. 50.4%; *P* < 0.001), vitamin B12 (80.0% vs. 51.2%; *P* < 0.001), and ferritin tests (60.0% vs. 39.2%; *P* = 0.004) were significantly higher in the micronutrient deficiency group while 25-OH-vitamin D test was lower than the non-deficiency group (83.8% vs. 94.4%; *P* = 0.013). The number of subjects found with deficiency of each micronutrient was 10 (12.5%) for folate, 20 (25.0%) for vitamin B12, 21 (26.3%) for 25-OH-vitamin D, and 45 (56.3%) for ferritin in the micronutrient deficiency group. Patients with CD exhibited a greater prevalence of micronutrient deficiency than those with UC or intestinal BD (82.5% vs. 11.3% vs. 6.3%; *P* = 0.003).

**Table 1:** Baseline characteristics between micro-nutrient deficiency group and non-deficiency group

Variables	Total (n = 205)	Micro-nutrient deficiency group (n = 80, 39.0%)	Non-deficiency group (n = 125, 61.0%)	*P-*value
Male sex	118 (57.6)	44 (55.0)	74 (59.2)	0.553
Age	34 (19–47)	27 (19–41)	37 (20–51)	0.003
The path of laboratory test				0.150
Outpatients clinic	191 (93.2)	72 (90.0)	119 (95.2)	
Hospitalization patients	14 (6.8)	8 (10.0)	6 (4.8)	
Number of micronutrient tests				
Folate	127 (62.0)	64 (80.0)	63 (50.4)	< 0.001
Vitamin B12	128 (62.4)	64 (80.0)	64 (51.2)	< 0.001
25-OH-vitamin D	184 (90.2)	67 (83.8)	117 (94.4)	0.013
Ferritin	97 (47.3)	48 (60.0)	49 (39.2)	0.004
Type of IBD				0.003
UC	49 (23.9)	9 (11.3)	40 (32.0)	
CD	143 (69.8)	66 (82.5)	77 (61.6)	
Intestinal BD	13 (6.3)	5 (6.3)	8 (6.4)	
Medications				
5-ASA	114 (55.6)	33 (41.3)	81 (64.8)	0.001
Steroids	12 (5.9)	3 (3.8)	9 (7.2)	0.305
Azathioprine	66 (32.2)	32 (40.0)	34 (27.2)	0.056
Anti-TNF	101 (49.3)	45 (56.3)	56 (44.8)	0.110
Methotrexate	33 (16.1)	11 (13.8)	22 (17.6)	0.464
6-MP	2 (1.0)	0 (0)	2 (1.6)	0.256
Others^a^	12 (5.9)	2 (2.5)	10 (8.0)	0.102
Supplementary medications				
Iron supplementation	77 (37.6)	43 (53.8)	34 (27.2)	< 0.001
Folate supplementation	50 (24.4)	26 (32.5)	24 (19.2)	0.031
Multivitamin	52 (25.4)	21 (26.3)	31 (24.8)	0.816
Calcium, Vitamin D supplementation	127 (62.0)	51 (63.7)	76 (60.8)	0.671
Vitamin B12 supplementation	43 (21.0)	30 (37.5)	13 (10.4)	< 0.001
Vitamin C supplementation	3 (1.5)	0 (0)	3 (2.4)	0.163
Zinc supplementation	7 (3.4)	3 (3.8)	4 (3.2)	0.832
Osteoporosis medication				
SERM	3 (1.5)	1 (1.3)	2 (1.6)	0.839
Bisphosphonate	14 (6.8)	1 (1.3)	13 (10.4)	0.011
Operation	65 (31.7)	33 (41.3)	32 (25.6)	0.019
Underlying disease				
Hypertension	4 (2.0)	1 (1.3)	3 (2.4)	0.561
Diabetes	1 (0.5)	0 (0)	1 (0.8)	0.423
Osteoporosis	38 (18.5)	13 (16.3)	25 (20.0)	0.500
Others^b^	46 (22.4)	19 (23.8)	27 (21.6)	0.719

Data are expressed as median (interquartile range, IQR) or n (%). *P-value for comparing patients with micronutrient deficiency group and non-deficiency group

IBD, Inflammatory bowel disease; UC, Ulcerative colitis; CD, Crohn’s disease; BD, Behçet’s disease; 5-ASA, 5-aminosalicylic acid; anti-TNF, anti-tumor necrosis factor; 6-MP, 6-mercaptopurine; SERM, Selective estrogen receptor modulators

^a^Mongersen, vedolizumab

^b^Myelodysplastic syndrome, Systemic lupus erythematosus, aortic valve disease, Bronchiolitis obliterans with organizing pneumonia, osteoarthritis, coronary artery occlusive disease, cancer

The median age was 34 years [IQR, 19–47], and 57.6% of the enrolled patients were male. In addition, most patients underwent laboratory testing in an outpatient clinic setting compared to an inpatient setting (93.2% vs. 6.8%; *P* = 0.150). There were no significant differences in most of the types of medications used by patients, but there were significantly more users of 5-aminosalicylic acid (5-ASA) in the non-deficient group (41.3% vs. 64.8%; *P* = 0.001). Micronutrient deficiency was higher in patients who underwent previous bowel surgery to treat the disease than in those who had not previously undergone disease-related surgery (41.3% vs. 25.6%; *P* = 0.019) (Table 1).

### Relative risk of micronutrient deficiency

Based on the univariate analysis, variables that were negatively associated with micronutrient deficiency with statistical significance included age (OR, 0.975; 95% CI 0.958–0.993; *P* = 0.006) and 5-ASA usage (OR, 0.381; 95% CI 0.214–0.679; *P* = 0.001). In addition, CD (compared to those with UC or intestinal BD) (OR, 3.810; 95% CI 1.721–8.430; *P* = 0.001) and previous disease-related surgical operations (OR, 2.041; 95% CI 1.120–3.717; *P* = 0.020) were significantly associated with micronutrient deficiency (Table 2). Based on the multivariate analysis assessing variables including male sex, age, the path of laboratory test, types of intestinal disease, medications, previous surgical operations, and other underlying diseases, CD (compared with UC or intestinal BD) (adjusted OR, 2.492; 95% CI 1.012–6.136; *P* = 0.047) and previous disease-related surgical operations (adjusted OR, 2.714; 95% CI 1.260–5.844; *P* = 0.011) were identified to be associated with higher risks of micronutrient deficiency. Conversely, age (adjusted OR, 0.973; 95% CI 0.949–0.998; *P* = 0.037) was negatively associated with micronutrient deficiency (Table 2).

**Table 2:** Relative risk of micro-nutrient deficiency

Variable	Uni-variate analysis		Multi-variate analysis	
	OR (95% CI)	*P*-value	Adjusted OR (95% CI)	*P*-value
Male sex	0.842 (0.478–1.485)	0.553	0.718 (0.375–1.376)	0.319
Age	0.975 (0.958–0.993)	0.006	0.973 (0.949–0.998)	0.037
The path of laboratory test				
Outpatients clinic	1.0 (Ref.)		1.0 (Ref.)	
Hospitalization patients	2.204 (0.735–6.608)	0.158	2.459 (0.715–8.461)	0.154
Type of disease				
UC	1.0 (Ref.)		1.0 (Ref.)	
CD	3.810 (1.721–8.430)	0.001	2.492 (1.012–6.136)	0.047
Intestinal BD	2.778 (0.734–10.513)	0.132	1.356 (0.284–6.479)	0.703
Medications				
5-ASA	0.381 (0.214–0.679)	0.001	0.548 (0.281–1.068)	0.077
Steroids	0.502 (0.132–1.914)	0.313		
Azathioprine	1.784 (0.983–3.238)	0.057		
Anti-TNF	1.584 (0.900–2.788)	0.111		
Methotrexate	0.746 (0.340–1.637)	0.465		
Others^a^	0.295 (0.063–1.383)	0.121		
Operation	2.041 (1.120–3.717)	0.020	2.714 (1.260–5.844)	0.011
Underlying disease				
Hypertension	0.515 (0.053–5.037)	0.568		
Osteoporosis	0.776 (0.371–1.624)	0.501	1.500 (0.699–3.217)	0.298
Others^b^	1.131 (0.579–2.206)	0.719	2.459 (0.715–8.461)	0.154

OR, odds ratio; CI, confidence interval; UC, Ulcerative colitis; CD, Crohn’s disease; BD, Behçet’s disease; 5-ASA, 5-aminosalicylic acid; anti-TNF, anti-tumor necrosis factor

^a^Mongersen, vedolizumab

^b^MDS, SLE, AR, BOOP, OA, Osteoporosis, CAOD, cancer

### Changes in micronutrient levels

**Fig. 2:**
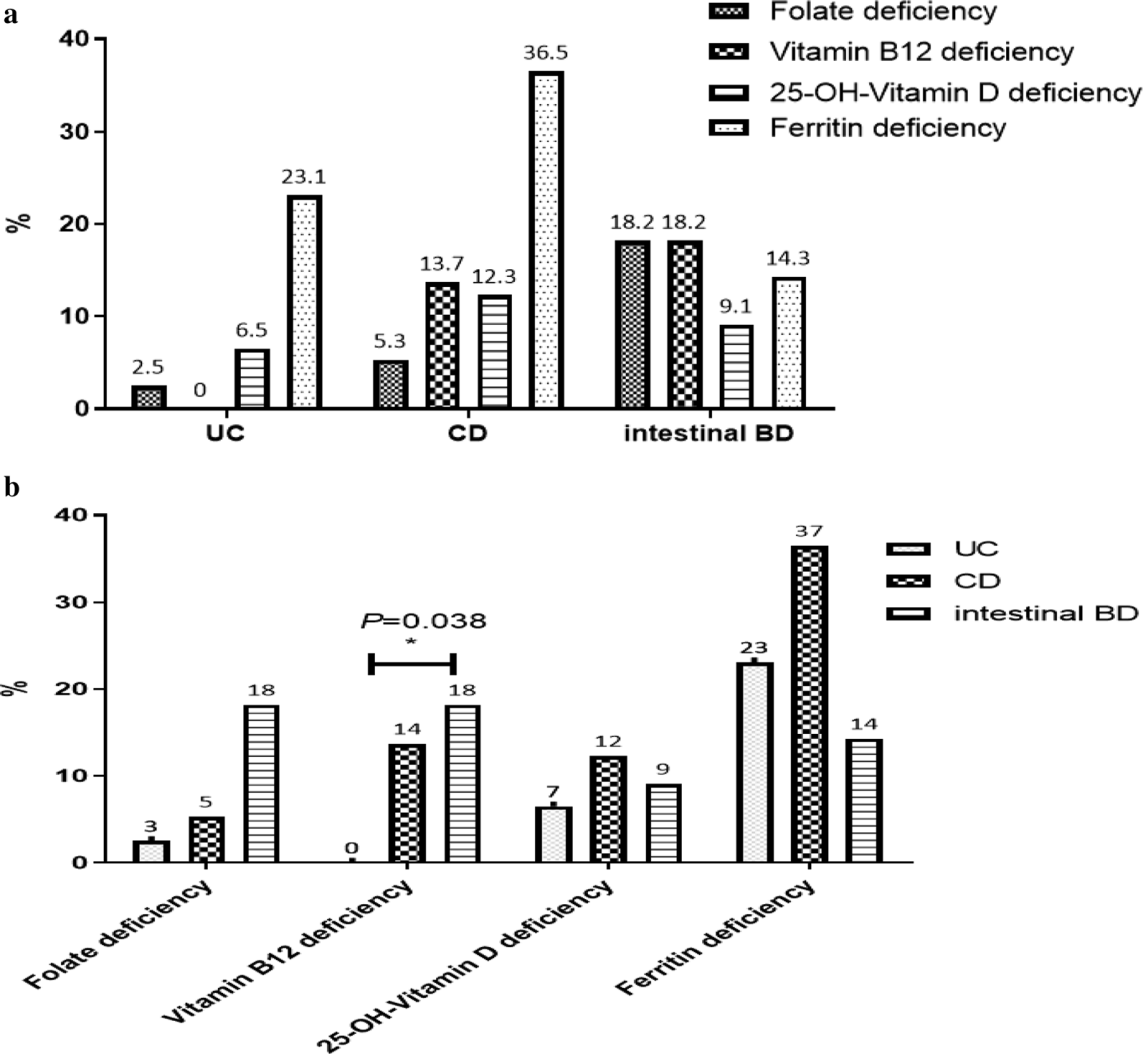
Frequency of micronutrient deficiency (folate, vitamin B12, 25-OH-vitamin D, and ferritin) for each disease, including ulcerative colitis (UC), Crohn’s disease (CD), and intestinal Behçet’s disease (BD)

Patients with UC and CD had the highest rates of ferritin deficiency compared to the other micronutrients assessed, including vitamin B12, folate, and 25-OH-vitamin D, while in those with intestinal BD, the deficiency did not vary among the micronutrients assessed. However, rates of vitamin B12 deficiency significantly differed among those with UC, CD, and intestinal BD (0% vs. 13.7% vs. 18.2%, respectively; *P* = 0.038) (Fig. 2).

**Fig. 3:**
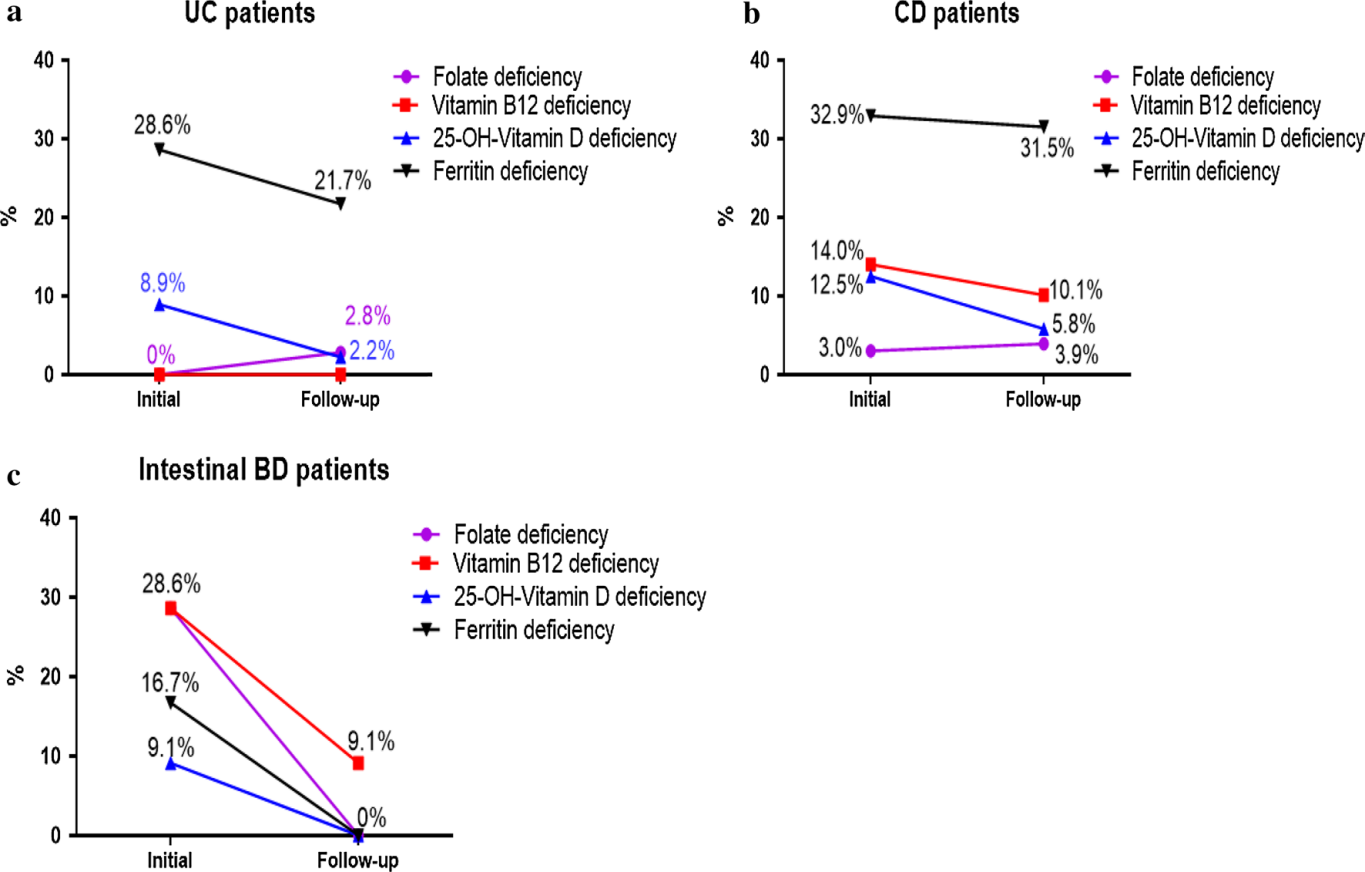
Changes in micronutrient deficiencies (folate, vitamin B12, 25-OH-vitamin D, and ferritin) between the initial and follow-up blood tests after six months in patients with ulcerative colitis (UC), Crohn’s disease (CD), and intestinal Behçet’s disease (BD)

We assessed changes from baseline at the first 6-month follow-up in terms of folate, vitamin B12, 25-OH-vitamin D, and ferritin levels in this study population. Initial laboratory tests in patients with UC showed ferritin and 25-OH-vitamin D deficiency, which had improved at follow-up testing after supplementation. In patients with CD, ferritin, vitamin B12, 25-OH-vitamin D, and folic acid deficiency occurred, and ferritin deficiency was the highest. Patients with intestinal BD had more deficiencies in folic acid and vitamin B12 than ferritin and 25-OH-vitamin D. In all cases, deficiencies were treated at the time of discovery, and after 6 months, all micronutrient deficiencies were reduced (Fig. 3).

## Discussion

The study found that micronutrient deficiency was high (39.0%) in patients with IBD and intestinal BD, and 83% of the deficiency group were CD patients. In IBD patients with CD or UC, ferritin deficiency was significant, as was 25-OH-vitamin D deficiency. However, patients with intestinal BD had more folic acid and vitamin B12 deficiencies than those with IBD, who had more ferritin and 25-OH-vitamin D deficiencies. In addition, young age, CD, and intestinal surgery were significantly associated with micronutrient deficiency.

Hwang et al. reviewed studies on micronutrient deficiency in patients with IBD and showed prevalence of micronutrients deficiencies. Among IBD patients, water-soluble vitamins deficiencies have been reported up to 11–78% [19, 23–25]; fat-soluble vitamins deficiencies to 22–90% [22, 23, 25–28]; and macro mineral deficiencies to 36–90% [15, 23, 24, 29–31]. In most studies, patients with CD showed a higher correlation with vitamin or mineral deficiencies than those with UC. Our study also showed a high prevalence (46.2%, 66 of 143 patients) of micronutrient deficiency in patients with CD. Most vitamins and minerals are absorbed in the proximal small intestine, and vitamin B12 is absorbed in the terminal ileum [9]. The distal ileum is also where bile acid absorption occurs, which is important for the absorption of fat and fat-soluble vitamins [9]. Therefore, micronutrient deficiency should be carefully observed in patients with CD, which is relatively invasive in the small intestine and frequently affects the terminal ileum. In addition, caution should also be exercised in cases of intestinal BD because it often manifests as an oval shape and causes deep ulcers in the ileocecal area [7]. Therefore, patients with intestinal BD also showed micronutrient deficiencies (38.46%, 5 of 13), especially vitamin B12 deficiency; there was a significant difference among patients with UC, CD, and intestinal BD, with the highest prevalence in patients with intestinal BD (UC, 0% vs. CD, 13.7% vs. intestinal BD, 18.2%; *P* = 0.038) (Fig. 2b). The mostly likely explanation could be due to the location of the intestine affected by the disease, as intestinal BD may be associated with high cobalamin (vitamin B12) deficiency rates. Therefore, 156 patients with CD and intestinal BD with similar invasion sites were analyzed separately, but there was no significant difference in the deficiency of micronutrients between CD and BD patients. However, our results showed that micronutrient deficiency was as high in patients with intestinal BD as well as in patients with CD. Further research is needed in a larger study group.

In patients with IBD, surgery is a significantly important factor affecting the likelihood of micronutrient deficiency. There have been reports of significantly lower vitamin D levels when CD affects the small intestine or when the small intestine is excised [32]. Active Crohn's ileitis and small bowel resection are reported to be risk factors for folic acid deficiency, as they can lead to malabsorption [19, 25]. In addition, Battat et al. reported that ileal resection greater than 30 cm was an associated risk factor for cobalamin deficiency in patients with CD [33]. When further analyzing the risk factors for cobalamin deficiency in our study population, disease-related surgical operation was found to be a significant associated risk factor (OR, 5.513; CI 1.829–16.613; *P* = 0.002; data not shown).

Our study is the first report on micronutrient deficiency in patients with intestinal BD and IBD. However, the study has several limitations. First, it is a retrospective cross-sectional study with selection bias. Because we examined patients who had been tested for a period of 1 year, we could not observe the cumulative effects of time to compare what the deficiencies had been before the start of the study and whether the deficiency occurred again after it ended, even though the deficiencies had been treated. This also showed a limit to the outcome of the assessment of patients with vitamin D deficiency. Even though our study used a lower vitamin D deficiency criterion (< 10 ng/mL) than other studies, many patients were already taking supplements (62.0%), which made it difficult to make a clear comparison. In addition, it may include cases in which several micronutrients are deficient in one patient with high risk of micronutrient deficiency. Furthermore, deficiency of certain micronutrients such as vitamin B12 can be affected depending on the location of the disease. For example, CD and intestinal BD patients are at high risk of vitamin B12 deficiency, while UC patients are relatively less likely to be affected. Therefore, there is a limitation with the possibility of selection bias. However, it is meaningful that it can analyze the risk of overall micronutrients deficiency. Second, we could not examine the relationship between disease activities. In particular, data on ESR (erythrocyte sedimentation rate) and CRP (C-reactive protein) levels, medical records of disease activity (CDAI [Crohn's disease activity index], Mayo score, DAIBD [Disease Activity Index for Intestinal Behcet's Disease]), and disease extent (Montreal classification) were insufficient for analysis. Most were outpatient clinic patients (93.2%), so we could not obtain accurate disease information based solely on the medical records. For example, 55.6% of the included patients were being treated with Pentasa (5-ASA) and 5.9% of patients were treated with steroid medications, suggesting most patients were not in the acute phase of disease progression. Thus, it may have been difficult to clearly compare differences in micronutrient deficiencies among those treated with various medications. Nevertheless, it was found that micronutrient deficiency was high in the outpatient population where the disease activity was not severe.

## Conclusions

In conclusion, in patients with IBD and intestinal BD, the incidence of micronutrient deficiency was high (39.0%). In addition, CD, a history of intestinal surgery, and young age were factors significantly associated with deficiency. Therefore, those with IBD, especially young patients with CD who have undergone bowel resection, should be observed more carefully to assess the need for supplementation to treat micronutrient deficiencies.

## Data Availability

Data is available from the corresponding author upon reasonable request.

## Abbreviations

IBD: Inflammatory bowel disease
CD: Crohn’s disease
UC: Ulcerative colitis
GI: Gastrointestinal
BD: Behçet’s disease
IQR: Interquartile range
ORs: Odds ratios
CIs: Confidence intervals
